# A distraction technique using reduction multi-axial screws for open reduction of high-grade lumbar posterior dislocation:a case report and literature review

**DOI:** 10.1186/s12891-019-2939-8

**Published:** 2019-11-15

**Authors:** Zhangzhe Zhou, Yimeng Wang, Zhiyong Sun, Xiaoyu Zhu, Zhonglai Qian

**Affiliations:** The Department of Orthopedic Surgery, The First Affiliated Hospital of Soochow University, 188 Shizi Street, Suzhou, 215006 Jiangsu China

**Keywords:** L3 vertebral fracture, Posterior dislocation, Reduction technique, Lumbar artery

## Abstract

**Background:**

L3 vertebral fractures with posterior dislocation are rare and usually secondary to high-energy trauma. To assess the outcome of a valuable distraction technique, using long-tail multiaxial pedicle screw which we have employed in reduction of L3 vertebral fracture with posterior dislocation, and emphasize the importance of preoperative blood vessel evaluation.

**Case presentation:**

A 47-year-old patient fell from a height of 4 m and was paralyzed. Computed tomography scan revealed a three-column ligamentous injury with posterior fracture-dislocation of the L3 vertebral body. Computed tomography angiography showed that the third lumbar artery was ruptured without active bleeding. The patient underwent posterior approach with reduction, transpedicular fixation, and posterolateral fusion with autologous bone graft. Finally, Vertebral reduction and sagittal balance were achieved and patients recovered well after operation.

**Conclusion:**

Preoperative blood vessel evaluation is very important to avoid massive bleeding during the surgery, and the standard technique which can achieve good reduction is easy to understand, perform, and is reproducible.

## Background

Lumbar burst fracture with dislocation is usually caused by high-energy trauma. It often occurs in high-activity and high-stress vertebral body parts of the spine, such as the junction of thoracolumbar vertebrae and lumbosacral vertebrae [1, 2]. There are several directions of lumbar dislocation, and posterior dislocation is rare and can lead to serious nerve injury [3–5]. After we carefully searched and consulted the literature, six similar cases were reported in the past 10 years [6–11] (Table 1). This study represents a case of L3 vertebral burst fracture with posterior dislocation, and the technique of reduction using reduction multi-axial screw with long tail is rarely reported for fracture with dislocation.

**Table 1:** Summary of cases about lumbar fracture with posterior dislocation in the literature

Author	Age/Sex	Level	Etiology	Operation	Sequelae
Robbins et al. [6]	23/F	L5-S1	Traffic accident	Internal fixation of L5-S1 pedicles and discectomy and posterior lumbar interbody fusion	Complete recovery
Yadav et al. [7]	7/M	L1-L2	Drag of rope	L2 reduction by a towel clip and stabilization by 5-mm loop rectangle and sublaminar wires with two levels above and below the affected segment	Incomplete recovery
Verhelst et al. [8]	6/F	L5-S1	Tractor crash	A laminectomy was performed from L4 to S1 and posterior instrumentation and grafting was performed by the USS Small Stature/ Pediatric spinal system	Incomplete recovery
Baron et al. [9]	20/M	L4-L5	Traffic accident	Posterior reduction with pedicle screw– and rod-augmented fusion from L3 to S1.	Incomplete recovery
Gabel et al. [10]	27/M	L5-S1	Traffic accident	Internal fixation of the spine from L2 to the sacroiliac joint and an interbody cage was placed at the L5 level	Incomplete recovery
Zhang et al. [11]	51/M	L4-L5	Fall accident	Depression, reduction, and fixation	No neurologic deficit

### Case presentation

#### Patient

A 47-year-old man presented with severe low back pain and dysfunction of lower limbs after falling from 4 m height. The patient was referred to our hospital from a junior hospital through emergency service 2 days later. The neurological examinations revealed that his Glasgow Coma Scale score was 15/15. The vital signs monitoring showed: BP: 80/50, HR:102, T:37.5 and RR: 18. In the initial examination, the lower extremity was 0/5 and that of proximal extremity was 1/5. Both lower limbs lost sensory and motor function, and no patellar and achilles tendon reflexes. The patient had urinary and bowel incontinence.

Routine blood tests showed that hemoglobin (HGB) was 78 g/L.

The computed tomography (CT) revealed a complete fracture-dislocation of the L3 vertebrae. The right transverse process of the L4 were fractured. Associated injuries were a fracture of the right 5-11th rib and a pulmonary contusion.

Considering of preventing hemorrhagic shock, the patient was given infusion of suspended erythrocyte 400 ml and plasma 300 ml immediately. The vital signs and hemodynamics of the patients stabilized after anti-shock treatment. CT angiography examination showed that the third lumbar artery was ruptured without active bleeding.

**Fig. 1:**
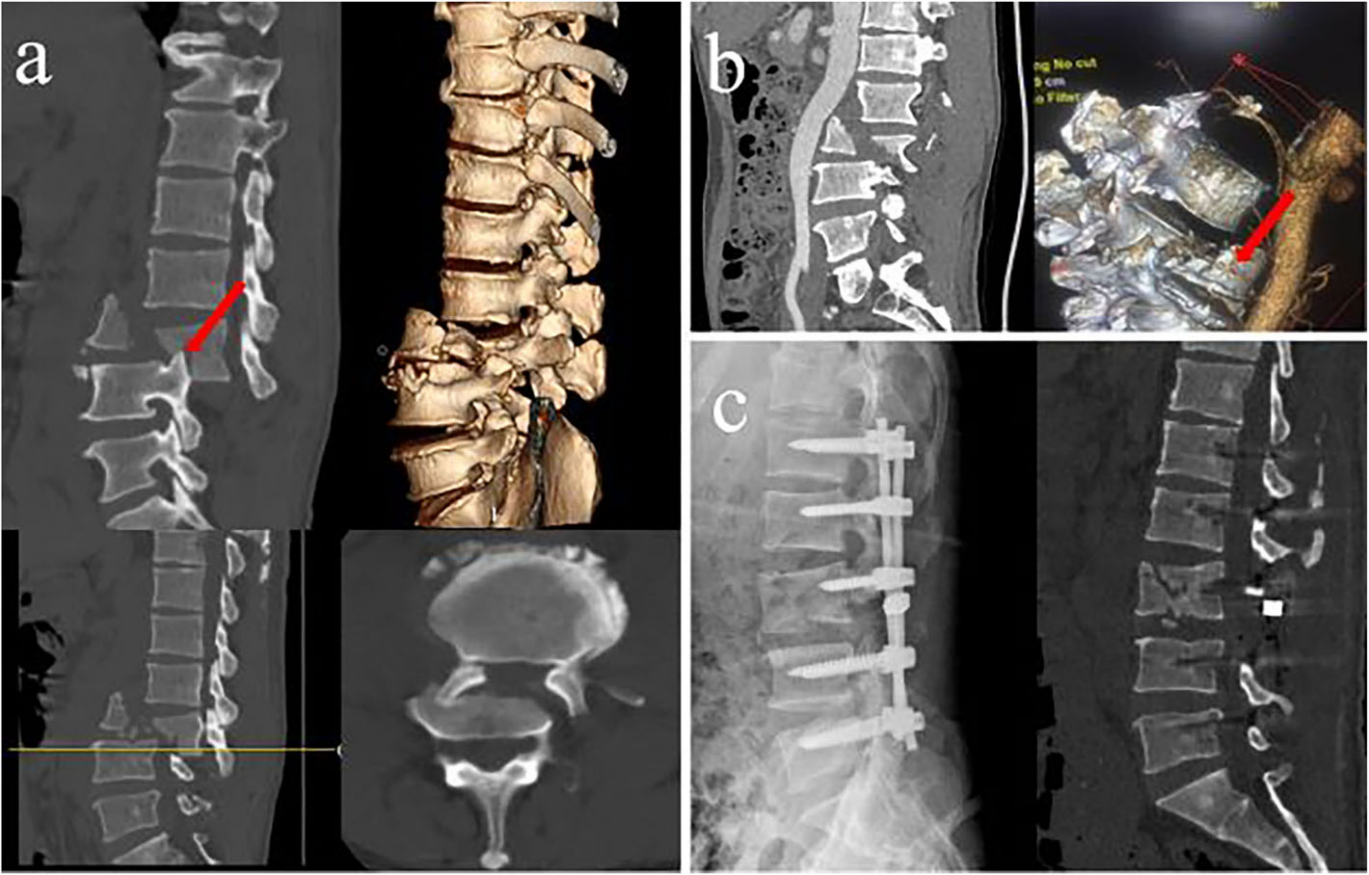
**a** Computed tomography (CT) showed L3 vertebral burst fracture with posterior dislocation, and fractured vertebral body formed articular process interlocking. **b** Computed tomography angiography (CTA) indicated fractured lumbar vertebra is adjacent to abdominal aorta and lumbar artery ruptured without active bleeding. **c** Postoperative X-ray and CT

The surgery was conducted 6 hours after referral to our hospital. (Fig. 1.)

### Surgical technique

#### Internal fixation instruments

Medtronic CD HORIZON Spinal System.

#### Patient position

Prone position (proper patient positioning may aid in achieving partial vertebral reduction and insertion of the pedicle screw).

#### Step

**Fig. 2:**
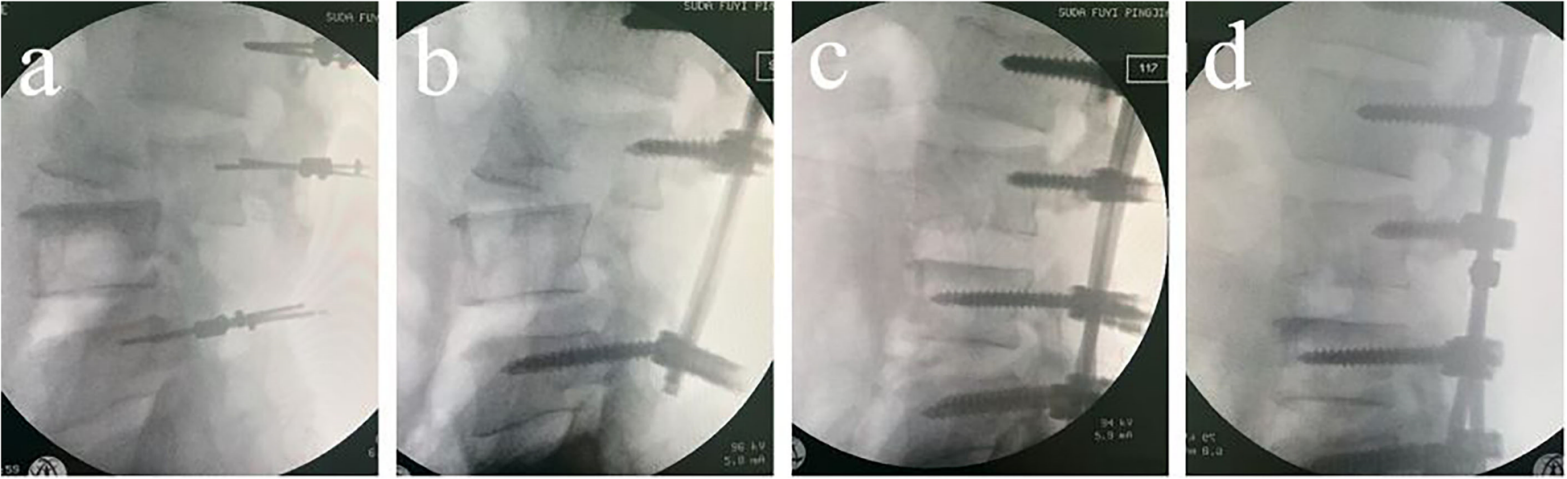
**a**, **b**, **c**, **d** Several key steps during the operation. **a** Guide pins were inserted at L1 L2 L3 L5 vertebrae. **b** The insertion points of the pedicle screws were exposed at the L4 vertebrae level, and part of the L3 vertebral plate was removed. **c** Pedicle screw was inserted into the L4 vertebrae and the L3–4 gap was distracted. **d** Pull-reduction of L3-L4 was achieved

1. Posterior midline approach—midline skin incision was performed the posterior vertebral elements including laminae, vertebral facets and transverse processes are exposed at the appropriate levels through peeling the paravertebral muscles close to the spinous process. The insertion point of the pedicle screw at the L4 vertebrae level which were under the lamina of L3 because of the dislocation, would be exposed later after the traction.

2. Guide pin and pedicles screws are inserted at L1 L2 L3 L5 vertebrae and the reduction multi-axial screws with long tails were inserted into the L5 vertebrae in order to provide enough space for the rod placement and pull-reduction. (Fig. 2 a).

3. Two straight rods were placed into the pedicle screws, after locking the tail cap of L1 L2 L3, with maintained distraction between L3 and L5, the surgeon locked the tail cap of L5, leading to the pull-reduction of L5 and expose of the insertion point of the pedicle screw at the L4 vertebrae level while part of the L3 vertebral plate could be removed to make the expose of the L4 insertion point easier. (Fig. 2 b).

4. A rod in one side was then removed and another long tail pedicle screw was inserted into the L4 vertebrae. Re-allied the removed rod through L1-L5, and lock the tap of L1 L2 L3 L5. Then the same procedure performed in the other side.

5. Increasing the sustained strength between L3–4 gradually after loosing the tap of L5 to distract the L3–4 gap, then the surgeon locked the tap of L4 screw to achieve pull-reduction of L3-L4. (Fig. 2 c).

6. The straight rods were replaced with the pre-bending rods one by one to recover the the lumbar physiological lordosis, Complete decompression of the cord with removal of spinous processes and bilateral laminectomies was performed at the injured level while part of the ligamentum flavum were left to prevent cerebrospinal fluid leakage.

7. A transverse cross-link was applied to provide additional strength and stability to the construct. Autologous graft materials, consisting of the leftover bones procured from laminectomy together were placed in the lateral gutter at the inter-transverse zones to facilitate and enhance fusion. (Fig. 2 d).

The blood loss was 800 ml and blood transfusion was 600 ml during the surgery.

Finally, the patient had no hemorrhagic shock symptoms and improved sensory function in perianal area and both proximal lower extremities after surgery. The lower extremity was 0/5 and that of proximal extremity was improved to 3/5. No patellar and Achilles tendon reflexes, and Feces and urine are still incontinent. Postoperative radiographs showed good reduction with maintained sagittal balance. After the operation, the patient was transferred to the rehabilitation department of our hospital for further treatment. After discharge, the patient went to the local hospital for functional exercise and was in the charge of a professional nursing team.

## Discussion

Studies have shown that traumatic thoracolumbar fractures with dislocation account for 3% of spine-related injuries. Because many patients died on the spot due to severe trauma, there were fewer patients with high-grade posterior lumbar dislocation [12]. Therefore, the treatment of lumbar fracture with high posterior dislocation is rarely reported. We report a case of L3 burst fracture and posterior dislocation with neurological impairment. According to the description of the patient and his family, the patient fell from a high altitude and fell to wood. We inferred the mechanism of this injury: hyperflexion and an upward shear force resulted in fracture and posterior dislocation of L3.

Computed tomography (CT) scan showed that L3 vertebral burst fracture with posterior dislocation and the pedicle screw insertion points of L4 vertebral were under L3 vertebral plate. Baron et al. reported a case of L4 vertebral fracture with posterior dislocation. The reduction was achieved by initial lengthening [9]. In severe fractures and dislocations, even with articular process interlocking, it is difficult to achieve perfect vertebral reduction by simply horizontal lengthening, and vertical pull-reduction of the dislocated vertebral body is necessary. Francis et al. reported a case of fracture with dislocation of the reduction site attempted by extracorporeal traction before operation [13]. This method has uncontrollable reduction direction and strength, as well as the risk of damage to blood vessels and nerves, so we did not attempt traction reduction, but direct surgery. Reduction with gradual lengthening (first L3-L5 and then L3-L4), has the advantages of gentle operation and less injury. Hadgaonkar et al. reported a case of manual reduction through short segment fixation of upper and lower vertebral bodies [14]. Because the operation is a direct manual reduction, there is a risk of nerve injury and the possibility that patients with strangulation cannot be unlocked. In this case, the key of fracture reduction is to lengthen and pull. Because of the long segmental fixation, with the help of professional instruments, the rods could be applied into the pedicle screws with long tails easily. Our method that the reduction multi-axial screws with long tails were locked slowly after lengthening achieved the satisfactory effect of pulling. In this case, if the L3-L5 vertebral body was fixed by a short rod in the first, there may be a high risk of pedicle destruction, even if the CT showed that the fractured L3 vertebral pedicle was intact. At the same time, the L3-L5 short rod maintained less strength than the long rod between L1-L3 and L5. In the operation, gap between L1-L3 and L5 was lengthened and pulled, and the insertion point of the pedicle screw at the L4 vertebrae level was exposed. Then, gap between L1-L3 and L4 was lengthened and pulled again after pedicle screws with long tails were inserted into the L4 vertebrae. Finally, the perfect reduction was achieved. The mechanical forces of lengthening and pulling is safe, effective, accurate, sustainable and controllable during the reduction process. The disadvantage of the technique is that it can not be used in patients with osteoporosis or pedicle destruction. In order to restore the stability of the spine, the anterior column of the spine should be fixed subsequently. However, in view of the patient’s condition, we did not adopt anterior column fixation: (1) The patient has achieved good reduction; (2) The paraplegia state before operation and the inability to get out of bed early after operation; (3) Avoid reoperation bleeding and prevent abdominal aorta injury; (4) Reduce costs.

Domenicucci et al. reported a case of lumbar fracture with dislocation and lumbar artery injury. Satisfactory results were achieved through endovascular embolization [15]. Therefore, CT angiography (CTA) was necessary for the patient who understand the condition of vascular injury. CTA results showed that the left third lumbar artery was interrupted continuously, but no pulsatile hemorrhage occurred. Combined with the vital signs of patient and no progressive bleeding, the indications for lumbar vertebral artery embolization were inadequate.

## Conclusion

Since L3 vertebral fractures with posterior dislocation are rare, it is difficult to compare different surgical approaches using large-scale or clinical controlled trials. Preoperative blood vessel evaluation is very important to avoid bleeding during the surgery. Long instrumentation with bony fusion is the recommended surgical method while the reduction is the key point. This standard technique which can achieve good reduction is easy to understand, perform, and is reproducible.

## Data Availability

All of the data appear within the manuscript.

## Abbreviations

CT: Computed Tomography
CTA: CT angiography
L3: the third lumbar
